# 2-Meth­oxy-3,4-diphenyl­phenol

**DOI:** 10.1107/S1600536809050855

**Published:** 2009-12-09

**Authors:** Ilia A. Guzei, Senthilvelan Annamalai, Howard E. Zimmerman

**Affiliations:** a Department of Chemistry, University of Wisconsin-Madison, 1101 University Ave, Madison, WI 53706, USA

## Abstract

The title compound, C_19_H_16_O_2_, was isolated as the major product after the solid-state photochemical reaction of 2-meth­oxy-4,4-diphenyl­cyclo­hexa-2,5-dienone. The dihedral angles between the central ring and pendant benzene rings are 60.76 (6) and 51.64 (6)°. The O—C vector of the meth­oxy group is almost perpendicular to the plane of the central ring as indicated by the C—C—O—C torsion angle of 94.89 (18)°. Hydrogen-bonded dimers are formed in the crystal structure *via* O—H⋯O inter­actions. The data were collected at room temperature on a Bruker SMART X2S diffractometer in the automated mode and processed manually thereafter.

## Related literature

For the characterization of reaction products, see: Frimer *et al.* (1994 ▶); Matoba *et al.* (1985 ▶). *Mogul* (Bruno *et al.*, 2002 ▶) was used for the geometrical analysis.
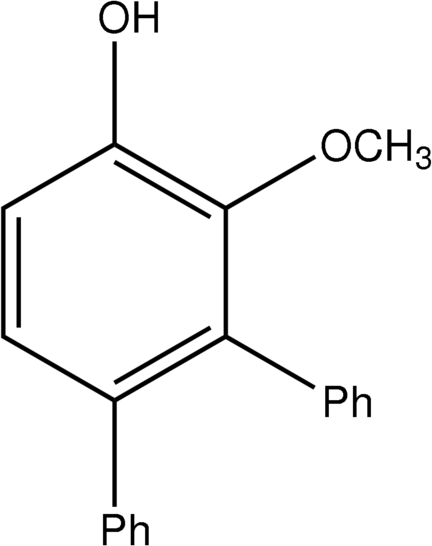


## Experimental

### 

#### Crystal data

C_19_H_16_O_2_
*M* *_r_* = 276.32
Monoclinic, 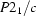

*a* = 14.312 (3) Å
*b* = 6.2585 (14) Å
*c* = 17.167 (4) Å
β = 102.930 (7)°
*V* = 1498.7 (6) Å^3^
*Z* = 4
Mo *K*α radiation
μ = 0.08 mm^−1^
*T* = 300 K
0.36 × 0.30 × 0.28 mm

#### Data collection

Bruker SMART X2S diffractometer
Absorption correction: multi-scan (*SADABS*; Bruker, 2007 ▶) *T*_min_ = 0.972, *T*_max_ = 0.978
13658 measured reflections
2645 independent reflections
1897 reflections with *I* > 2σ(*I*)
*R*_int_ = 0.040

#### Refinement

*R*[*F* ^2^ > 2σ(*F* ^2^)] = 0.039
*wR*(*F* ^2^) = 0.129
*S* = 1.01
2645 reflections
192 parameters
H-atom parameters constrained
Δρ_max_ = 0.16 e Å^−3^
Δρ_min_ = −0.19 e Å^−3^

### 

Data collection: *GIS* (Bruker, 2009 ▶); cell refinement: *SAINT* (Bruker, 2007 ▶); data reduction: *SAINT*; program(s) used to solve structure: *SHELXTL* (Sheldrick, 2008 ▶); program(s) used to refine structure: *SHELXTL* and *OLEX2* (Dolomanov *et al.*, 2009 ▶); mol­ecular graphics: *SHELXTL* (Sheldrick, 2008 ▶); software used to prepare material for publication: local programs (Guzei, 2007 ▶) and *publCIF* (Westrip, 2009 ▶).

## Supplementary Material

Crystal structure: contains datablocks global, I. DOI: 10.1107/S1600536809050855/tk2575sup1.cif

Structure factors: contains datablocks I. DOI: 10.1107/S1600536809050855/tk2575Isup2.hkl

Additional supplementary materials:  crystallographic information; 3D view; checkCIF report

## Figures and Tables

**Table 1:** Hydrogen-bond geometry (Å, °)

*D*—H⋯*A*	*D*—H	H⋯*A*	*D*⋯*A*	*D*—H⋯*A*
O2—H2⋯O1^i^	0.82	2.21	2.9043 (18)	142

Symmetry code: (i) 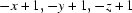.

**Fig. 1.**
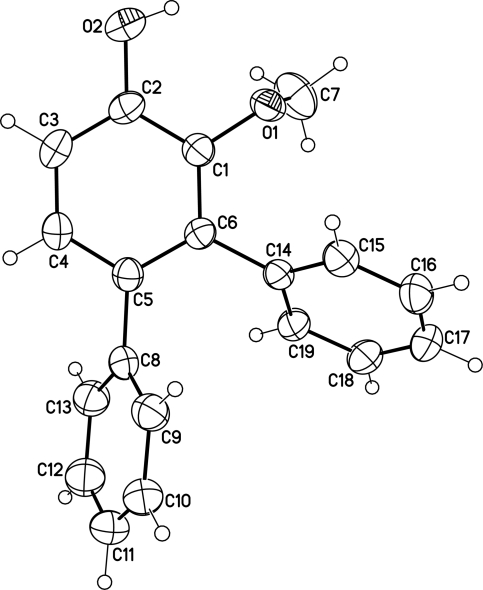
Molecular structure of (I) showing the atom labelling and displacement ellipsoids at the 50% probability level.

**Fig. 2.**
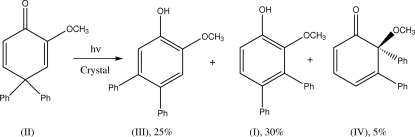
The formation of the title compound.

## supplementary crystallographic information

### Comment

The title compound (I), Fig. 1, was isolated as the major product during our studies of the solid state photochemical behavior of 2-methoxy-4,4-diphenyl-2,5-cyclohexadienone as described in the Experimental.

All bond distances and angles in (I) fall in the expected ranges according to the *Mogul* structural check (Bruno, *et al.*, 2002). The dihedral angles between the C1—C6 ring and rings C8—C13 and C14—C19 are 60.76 (6)° and 51.64 (6)°, respectively. The O1—C7 vector of the methoxy group is almost perpendicular to the plane of the C1—C6 ring as indicated by the dihedral angle C2—C1—O1—C7 of 94.89 (18)°. Compound (I) forms hydrogen-bonded dimers in the solid state. The graph set notation for the dimers connected by O2—H2···O1 hydrogen bonds is R_2_^2^(10).

### Experimental

Solid state photolysis of 2-methoxy-4,4-diphenyl-2,5-cyclohexadienone (II) was studied as follows.

A thin film of (II) (0.30 g, 0.001 mol) was irradiated under nitrogen for 18 h through a CuSO_4_ filter with a 400 watt medium pressure mercury lamp. The lamp was surrounded by a water-cooled immersion jacket and a cylindrical flask. The film of (II) was deposited on the inner wall of the outer flask (4 cm from the lamp) by slow evaporation of its dichloromethane solution and was dried before photolysis under nitrogen for 2 h. The resulting orange solid was chromatographed (silica gel 2.5 cm *x* 37 cm), eluted with hexane-CH_2_Cl_2_ (2:3) to give the following: band 1, 2-methoxy-4,5-diphenylphenol (III); band 2, 2-methoxy-3,4-diphenylphenol (I), band 3, 6-methoxy-5,6-diphenyl-2,4-cyclohexadienone (IV). The unreacted (band 4) cyclohexadienone (II) (0.10 g, 33%) was separated by using CH_2_Cl_2_—CH_3_OH (9:1) elution (Scheme 1).

2-Methoxy-4,5-diphenylphenol (III) (Band 1): Recrystallization from ether-hexane gave (75 mg, 25%) colorless crystals, mp 419–421 K (Literature mp 422–423 K (Frimer *et al.*, 1994)). ^1^H NMR (CDCl_3_, 300 MHz): δ 3.95 (s, 3H), 5.62 (s, 1H), 6.91 (s, 1H), 7.02 (s, 1H), 7.10–7.21 (m, 10H) p.p.m.; ^13^C NMR (CDCl_3_, 75 MHz) δ 56.3, 113.3, 116.9, 126.4, 128.0, 128.1, 130.2, 130.2, 133.3, 134.1, 141.6, 142.0, 145.1, 146.1 p.p.m.

2-Methoxy-3,4-diphenylphenol (I) (Band 2): Recrystallization from CH_2_Cl_2_-hexane gave (90 mg, 30%) colorless crystals, mp 417–419 K. ^1^H NMR (CDCl_3_, 300 MHz): δ 3.29 (s, 3H), 5.88 (s, 1H), 6.99–7.04 (m, 3H), 7.08–7.17 (m, 6H), 7.20–7.24 (m, 3H) p.p.m.; ^13^C NMR (CDCl_3_, 75 MHz) δ 60.7, 114.5, 126.2, 126.8, 127.1, 127.8, 128.0, 130.2, 131.1, 133.8, 134.5, 136.3, 141.3, 145.3, 148.4 p.p.m.

6-Methoxy-5,6-diphenyl-2,4-cyclohexadienone (IV) (Band 3): This was recrystallized from CH_2_Cl_2_-hexane mixture. Yield = 15 mg, 5%; mp 467–469 K (Literature mp 469–471 K (Matoba *et al.*,1985). ^1^H NMR (CDCl_3_, 300 MHz): δ 3.66 (s, 3H, OCH_3_), 5.56 (d, J = 7.8 Hz, 1H), 5.88 (d, J = 9.9 Hz, 1H), 7.15–7.29 (m, 11H) p.p.m.; ^13^C NMR (CDCl_3_, 75 MHz) δ 56.2, 69.5, 94.5, 118.8, 127.6, 128.3, 129.9, 141.0, 143.5, 171.2, 201.2 p.p.m.

### Refinement

All H-atoms were placed in idealized locations (N—H = 0.82 Å and C—H = 0.93–0.96 Å) and refined as riding with *U*_iso_(H) = 1.2-1.5*U*_eq_(bearing atom).

### Crystal data

**Table:**

C_19_H_16_O_2_	*F*(000) = 584
*M**_r_* = 276.32	*D*_x_ = 1.225 Mg m^−^^3^
Monoclinic, *P*2_1_/*c*	Mo *K*α radiation, λ = 0.71073 Å
Hall symbol: -P 2ybc	Cell parameters from 999 reflections
*a* = 14.312 (3) Å	θ = 2.4–25.0°
*b* = 6.2585 (14) Å	µ = 0.08 mm^−^^1^
*c* = 17.167 (4) Å	*T* = 300 K
β = 102.930 (7)°	Block, colourless
*V* = 1498.7 (6) Å^3^	0.36 × 0.30 × 0.28 mm
*Z* = 4	

### Data collection

**Table:**

Bruker SMART X2S diffractometer	2645 independent reflections
Radiation source: micro-focus sealed tube	1897 reflections with *I* > 2σ(*I*)
doubly curved silicon crystal	*R*_int_ = 0.040
ω scans	θ_max_ = 25.0°, θ_min_ = 2.4°
Absorption correction: multi-scan (*SADABS*; Bruker, 2007)	*h* = −17→17
*T*_min_ = 0.972, *T*_max_ = 0.978	*k* = −7→7
13658 measured reflections	*l* = −20→20

### Refinement

**Table:**

Refinement on *F*^2^	Primary atom site location: structure-invariant direct methods
Least-squares matrix: full	Secondary atom site location: difference Fourier map
*R*[*F*^2^ > 2σ(*F*^2^)] = 0.039	Hydrogen site location: inferred from neighbouring sites
*wR*(*F*^2^) = 0.129	H-atom parameters constrained
*S* = 1.01	*w* = 1/[σ^2^(*F*_o_^2^) + (0.0877*P*)^2^] where *P* = (*F*_o_^2^ + 2*F*_c_^2^)/3
2645 reflections	(Δ/σ)_max_ < 0.001
192 parameters	Δρ_max_ = 0.16 e Å^−^^3^
0 restraints	Δρ_min_ = −0.19 e Å^−^^3^

### Special details

**Table:**

Geometry. All e.s.d.'s (except the e.s.d. in the dihedral angle between two l.s. planes) are estimated using the full covariance matrix. The cell e.s.d.'s are taken into account individually in the estimation of e.s.d.'s in distances, angles and torsion angles; correlations between e.s.d.'s in cell parameters are only used when they are defined by crystal symmetry. An approximate (isotropic) treatment of cell e.s.d.'s is used for estimating e.s.d.'s involving l.s. planes.
Refinement. Refinement of *F*^2^ against ALL reflections. The weighted *R*-factor *wR* and goodness of fit *S* are based on *F*^2^, conventional *R*-factors *R* are based on *F*, with *F* set to zero for negative *F*^2^. The threshold expression of *F*^2^ > σ(*F*^2^) is used only for calculating *R*-factors(gt) *etc*. and is not relevant to the choice of reflections for refinement. *R*-factors based on *F*^2^ are statistically about twice as large as those based on *F*, and *R*- factors based on ALL data will be even larger.

### Fractional atomic coordinates and isotropic or equivalent isotropic displacement parameters (Å^2^)

**Table:**

	*x*	*y*	*z*	*U*_iso_*/*U*_eq_	
O1	0.40221 (7)	0.44465 (18)	0.45356 (6)	0.0466 (3)	
O2	0.55156 (8)	0.1831 (2)	0.44401 (8)	0.0630 (4)	
H2	0.5431	0.2608	0.4803	0.095*	
C1	0.39866 (10)	0.3357 (2)	0.38259 (9)	0.0388 (4)	
C2	0.47494 (10)	0.2000 (3)	0.38019 (10)	0.0443 (4)	
C3	0.47283 (12)	0.0780 (3)	0.31276 (11)	0.0501 (5)	
H3	0.5228	−0.0155	0.3110	0.060*	
C4	0.39596 (11)	0.0956 (3)	0.24786 (10)	0.0453 (4)	
H4	0.3945	0.0107	0.2031	0.054*	
C5	0.32054 (10)	0.2373 (2)	0.24768 (9)	0.0388 (4)	
C6	0.32128 (10)	0.3600 (2)	0.31656 (9)	0.0352 (4)	
C7	0.35107 (16)	0.3378 (4)	0.50515 (13)	0.0751 (6)	
H7A	0.2843	0.3301	0.4795	0.113*	
H7C	0.3586	0.4158	0.5543	0.113*	
H7B	0.3761	0.1960	0.5162	0.113*	
C8	0.24411 (10)	0.2612 (3)	0.17289 (10)	0.0413 (4)	
C9	0.22906 (13)	0.4551 (3)	0.13291 (11)	0.0532 (5)	
H9	0.2650	0.5736	0.1542	0.064*	
C10	0.16117 (15)	0.4750 (4)	0.06154 (12)	0.0648 (6)	
H10	0.1522	0.6063	0.0355	0.078*	
C11	0.10739 (14)	0.3023 (4)	0.02930 (12)	0.0668 (6)	
H11	0.0620	0.3157	−0.0185	0.080*	
C12	0.12096 (13)	0.1098 (4)	0.06801 (13)	0.0660 (6)	
H12	0.0843	−0.0074	0.0463	0.079*	
C13	0.18878 (12)	0.0874 (3)	0.13916 (11)	0.0532 (5)	
H13	0.1974	−0.0447	0.1646	0.064*	
C14	0.24119 (10)	0.5068 (2)	0.32365 (9)	0.0368 (4)	
C15	0.25989 (12)	0.7172 (3)	0.34987 (10)	0.0464 (4)	
H15	0.3223	0.7691	0.3600	0.056*	
C16	0.18630 (14)	0.8488 (3)	0.36086 (11)	0.0580 (5)	
H16	0.1996	0.9883	0.3785	0.070*	
C17	0.09338 (14)	0.7743 (3)	0.34579 (12)	0.0634 (5)	
H17	0.0441	0.8626	0.3538	0.076*	
C18	0.07375 (12)	0.5682 (3)	0.31880 (12)	0.0577 (5)	
H18	0.0110	0.5179	0.3081	0.069*	
C19	0.14693 (11)	0.4360 (3)	0.30755 (10)	0.0456 (4)	
H19	0.1328	0.2975	0.2889	0.055*	

### Atomic displacement parameters (Å^2^)

**Table:**

	*U*^11^	*U*^22^	*U*^33^	*U*^12^	*U*^13^	*U*^23^
O1	0.0403 (6)	0.0606 (8)	0.0379 (6)	−0.0037 (5)	0.0068 (5)	−0.0070 (5)
O2	0.0436 (7)	0.0751 (10)	0.0632 (9)	0.0138 (6)	−0.0030 (6)	−0.0042 (7)
C1	0.0354 (8)	0.0435 (9)	0.0389 (9)	−0.0043 (6)	0.0114 (7)	−0.0017 (7)
C2	0.0323 (8)	0.0502 (10)	0.0499 (10)	0.0019 (7)	0.0081 (7)	0.0050 (8)
C3	0.0415 (9)	0.0498 (11)	0.0617 (12)	0.0109 (7)	0.0172 (8)	0.0012 (8)
C4	0.0473 (9)	0.0456 (10)	0.0466 (10)	0.0035 (7)	0.0184 (8)	−0.0033 (8)
C5	0.0401 (8)	0.0402 (9)	0.0387 (9)	−0.0003 (6)	0.0141 (7)	0.0010 (7)
C6	0.0315 (7)	0.0378 (8)	0.0377 (9)	−0.0010 (6)	0.0106 (6)	0.0030 (7)
C7	0.0934 (15)	0.0886 (16)	0.0522 (12)	−0.0144 (12)	0.0352 (11)	−0.0028 (11)
C8	0.0425 (8)	0.0460 (10)	0.0377 (9)	0.0045 (7)	0.0138 (7)	−0.0020 (7)
C9	0.0626 (11)	0.0505 (11)	0.0455 (10)	0.0042 (8)	0.0099 (8)	0.0000 (8)
C10	0.0763 (14)	0.0673 (13)	0.0487 (12)	0.0224 (11)	0.0096 (10)	0.0073 (10)
C11	0.0560 (11)	0.0907 (16)	0.0481 (12)	0.0149 (11)	−0.0003 (9)	−0.0069 (11)
C12	0.0487 (10)	0.0768 (15)	0.0670 (14)	−0.0026 (9)	0.0013 (9)	−0.0157 (11)
C13	0.0493 (10)	0.0531 (11)	0.0555 (11)	−0.0016 (8)	0.0082 (8)	−0.0036 (9)
C14	0.0367 (8)	0.0420 (9)	0.0320 (8)	0.0018 (6)	0.0081 (6)	−0.0003 (7)
C15	0.0510 (9)	0.0437 (10)	0.0446 (10)	−0.0018 (7)	0.0111 (7)	0.0000 (8)
C16	0.0765 (13)	0.0435 (11)	0.0527 (11)	0.0110 (9)	0.0116 (10)	−0.0052 (9)
C17	0.0588 (11)	0.0742 (14)	0.0566 (12)	0.0296 (10)	0.0112 (9)	−0.0059 (10)
C18	0.0369 (9)	0.0767 (14)	0.0582 (12)	0.0099 (8)	0.0075 (8)	−0.0063 (10)
C19	0.0365 (8)	0.0506 (10)	0.0489 (10)	0.0018 (7)	0.0075 (7)	−0.0051 (8)

### Geometric parameters (Å, °)

**Table:**

O1—C1	1.3871 (18)	C9—H9	0.9300
O1—C7	1.434 (2)	C10—C11	1.370 (3)
O2—C2	1.3698 (19)	C10—H10	0.9300
O2—H2	0.8200	C11—C12	1.368 (3)
C1—C2	1.391 (2)	C11—H11	0.9300
C1—C6	1.405 (2)	C12—C13	1.387 (3)
C2—C3	1.382 (2)	C12—H12	0.9300
C3—C4	1.384 (2)	C13—H13	0.9300
C3—H3	0.9300	C14—C19	1.388 (2)
C4—C5	1.397 (2)	C14—C15	1.398 (2)
C4—H4	0.9300	C15—C16	1.383 (2)
C5—C6	1.408 (2)	C15—H15	0.9300
C5—C8	1.496 (2)	C16—C17	1.378 (3)
C6—C14	1.495 (2)	C16—H16	0.9300
C7—H7A	0.9600	C17—C18	1.378 (3)
C7—H7C	0.9600	C17—H17	0.9300
C7—H7B	0.9600	C18—C19	1.382 (2)
C8—C9	1.387 (2)	C18—H18	0.9300
C8—C13	1.393 (2)	C19—H19	0.9300
C9—C10	1.389 (3)		
			
C1—O1—C7	112.95 (13)	C10—C9—H9	119.5
C2—O2—H2	109.5	C11—C10—C9	120.29 (19)
O1—C1—C2	116.83 (13)	C11—C10—H10	119.9
O1—C1—C6	121.54 (13)	C9—C10—H10	119.9
C2—C1—C6	121.63 (14)	C12—C11—C10	119.52 (18)
O2—C2—C3	119.41 (14)	C12—C11—H11	120.2
O2—C2—C1	121.10 (15)	C10—C11—H11	120.2
C3—C2—C1	119.49 (15)	C11—C12—C13	120.80 (19)
C2—C3—C4	119.66 (15)	C11—C12—H12	119.6
C2—C3—H3	120.2	C13—C12—H12	119.6
C4—C3—H3	120.2	C12—C13—C8	120.53 (17)
C3—C4—C5	121.82 (16)	C12—C13—H13	119.7
C3—C4—H4	119.1	C8—C13—H13	119.7
C5—C4—H4	119.1	C19—C14—C15	118.20 (14)
C4—C5—C6	118.94 (14)	C19—C14—C6	121.10 (14)
C4—C5—C8	118.79 (14)	C15—C14—C6	120.66 (13)
C6—C5—C8	122.20 (13)	C16—C15—C14	120.53 (16)
C1—C6—C5	118.38 (13)	C16—C15—H15	119.7
C1—C6—C14	118.76 (13)	C14—C15—H15	119.7
C5—C6—C14	122.79 (13)	C17—C16—C15	120.36 (17)
O1—C7—H7A	109.5	C17—C16—H16	119.8
O1—C7—H7C	109.5	C15—C16—H16	119.8
H7A—C7—H7C	109.5	C18—C17—C16	119.70 (16)
O1—C7—H7B	109.5	C18—C17—H17	120.2
H7A—C7—H7B	109.5	C16—C17—H17	120.2
H7C—C7—H7B	109.5	C17—C18—C19	120.23 (17)
C9—C8—C13	117.81 (16)	C17—C18—H18	119.9
C9—C8—C5	120.99 (15)	C19—C18—H18	119.9
C13—C8—C5	121.14 (15)	C18—C19—C14	120.97 (16)
C8—C9—C10	121.06 (18)	C18—C19—H19	119.5
C8—C9—H9	119.5	C14—C19—H19	119.5
			
C7—O1—C1—C2	−94.89 (18)	C6—C5—C8—C13	−123.15 (17)
C7—O1—C1—C6	84.55 (18)	C13—C8—C9—C10	−0.1 (3)
O1—C1—C2—O2	−2.8 (2)	C5—C8—C9—C10	177.00 (16)
C6—C1—C2—O2	177.77 (14)	C8—C9—C10—C11	0.1 (3)
O1—C1—C2—C3	176.31 (14)	C9—C10—C11—C12	0.1 (3)
C6—C1—C2—C3	−3.1 (2)	C10—C11—C12—C13	−0.3 (3)
O2—C2—C3—C4	−179.50 (15)	C11—C12—C13—C8	0.4 (3)
C1—C2—C3—C4	1.4 (2)	C9—C8—C13—C12	−0.2 (2)
C2—C3—C4—C5	1.3 (3)	C5—C8—C13—C12	−177.25 (16)
C3—C4—C5—C6	−2.3 (2)	C1—C6—C14—C19	−125.28 (16)
C3—C4—C5—C8	174.84 (15)	C5—C6—C14—C19	51.8 (2)
O1—C1—C6—C5	−177.28 (13)	C1—C6—C14—C15	52.1 (2)
C2—C1—C6—C5	2.1 (2)	C5—C6—C14—C15	−130.82 (16)
O1—C1—C6—C14	−0.1 (2)	C19—C14—C15—C16	1.3 (2)
C2—C1—C6—C14	179.32 (14)	C6—C14—C15—C16	−176.18 (15)
C4—C5—C6—C1	0.6 (2)	C14—C15—C16—C17	−0.2 (3)
C8—C5—C6—C1	−176.46 (13)	C15—C16—C17—C18	−0.7 (3)
C4—C5—C6—C14	−176.52 (13)	C16—C17—C18—C19	0.6 (3)
C8—C5—C6—C14	6.5 (2)	C17—C18—C19—C14	0.5 (3)
C4—C5—C8—C9	−117.18 (18)	C15—C14—C19—C18	−1.4 (2)
C6—C5—C8—C9	59.8 (2)	C6—C14—C19—C18	176.05 (15)
C4—C5—C8—C13	59.8 (2)		

### Hydrogen-bond geometry (Å, °)

**Table:**

*D*—H···*A*	*D*—H	H···*A*	*D*···*A*	*D*—H···*A*
O2—H2···O1^i^	0.82	2.21	2.9043 (18)	142

Symmetry codes: (i) −*x*+1, −*y*+1, −*z*+1.
